# Dioxygen, an unexpected carbonic anhydrase ligand

**DOI:** 10.1080/14756366.2018.1475371

**Published:** 2018-05-28

**Authors:** Marta Ferraroni, Roberto Gaspari, Andrea Scozzafava, Andrea Cavalli, Claudiu T. Supuran

**Affiliations:** a Dipartimento di Chimica, Università di Firenze, Sesto Fiorentino (FI), Italia;; b Istituto Italiano di Tecnologia, Genova, Italia;; c Dipartimento di Farmacia e Biotecnologie, Università di Bologna, Bologna, Italia;; d Dipartimento NEUROFARBA – Pharmaceutical and Nutraceutical Section, Sesto Fiorentino (FI), Italia

**Keywords:** Carbonic anhydrase, oxygen, crystallography, molecular dynamics

## Abstract

Carbonic anhydrases (CAs, EC 4.2.1.1) are ubiquitous metalloenzymes, grouped into seven different classes, which catalyze the reaction of CO_2_ hydration to bicarbonate and protons. All of the fifteen human isoforms reported to date belong to the α-class and contain zinc as a cofactor. The structure of human Zn,Cu-CA II has been solved which contains a copper ion bound at its N-terminal, coordinated to His4 and His64. In the active site a dioxygen molecule is coordinated to the zinc ion. Since dioxygen is a rather unexpected CA ligand, molecular dynamics (MD) simulations were performed which suggested a superoxide character of the zinc bound O_2_.

## 1. Introduction

One of the most abundant zinc enzymes in the blood is carbonic anhydrase (CA, EC 4.2.1.1), which catalyzes a simple but essential reaction in all life kingdoms, CO_2_ hydration to bicarbonate and protons^1–4^. This reaction, or the three chemical entities involved in it, carbon dioxide, bicarbonate and protons, are important for the pH regulation and homeostasis of the organism, CO_2_ and HCO_3_^−^ transport in several biosynthetic processes, for the production of body fluids, bone resorption, tumorigenicity, and other physiological processes in vertebrates, whereas in some bacteria, plants and algae they are involved in photosynthetic processes^5–7^.

**Figure 1.**
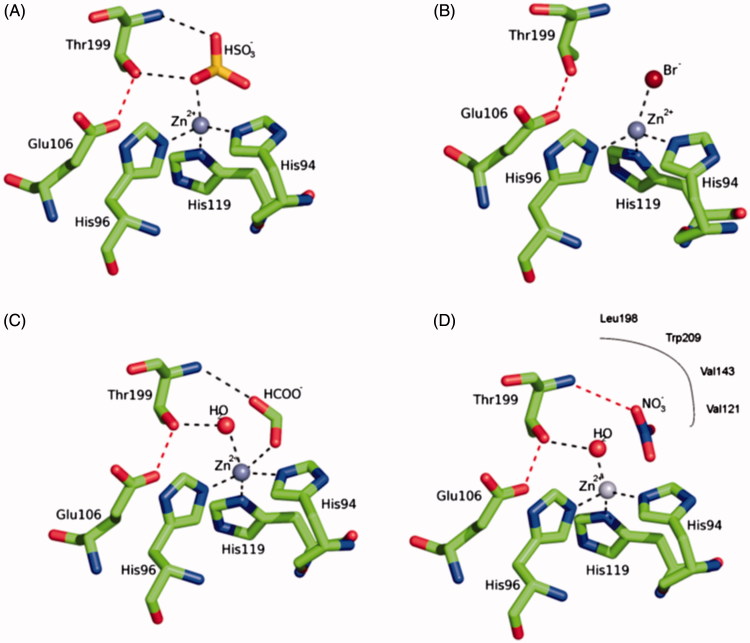
Structure of hCA II complexed with (A) bisulfite (tetrahedral geometry of Zn(II)), (B) bromide (distorted tetrahedral geometry of Zn(II)), (C) formate (trigonal bipyramidal geometry of Zn(II)) and (D) nitrate (inhibitor non-coordinated to the zinc)^10–13^. The three protein zinc ligands (His94, 96 and 119) as well as the other two amino acid residues involved in the catalytic mechanism and binding of inhibitors, Glu106 and Thr199, are also evidenced.^1^^,^^10^.

The catalytic mechanism of CAs is understood in detail^1–3^. In all CA classes known to date (α-, β-, γ-, δ-, ζ-, η- and θ-CAs) a metal hydroxide species (L_3_-M^2+^-OH^-^) of the enzyme is the catalytically active species, acting as a strong nucleophile (at neutral pH) on the CO_2_ molecule bound in a hydrophobic pocket nearby^1–6^. This hydroxide species is generated from a water coordinated to the metal ion, which is found at the bottom of the active site cavity. The active center normally comprises M(II) ions in tetrahedral geometry, with three protein ligands (L) in addition to the water molecule/hydroxide ion, although Zn(II) or Co(II) were also observed in trigonal bipyramidal or octahedral coordination geometries, at least in γ-CAs^7^. In many enzymes, generation of the hydroxide species from the metal-coordinated water one, is the rate determining step of the catalytic turnover, which for some α- and ζ-CAs achieves k_cat_/K_M_ values >10^8^ M^−1^ × s^−1^, making CAs among the most effective catalysts known in nature^1–3^. The metal ion ligands are three His residues in α-, γ-, and δ-CAs or one His and two Cys residues in β- and ζ-CAs^1–7^ .The inhibition and activation of CAs are well understood processes, with most types of inhibitors binding to the metal center^8^, whereas the activators bind at the entrance of the active site cavity where they participate in the proton shuttling between the metal-coordinated water molecule and the environment^9^. Inorganic simple anions are an important class of CA inhibitors (CAIs)^10^. Both metal-complexing anions (such as cyanide, thiocyanate, hydrogen sulfide, etc.) as well as anions showing less affinity for metal ions in solution (such as nitrate, bisulfite, sulfate, sulfamate and sulfamidate) are known to inhibit these metalloenzymes^10^, and for many of them detailed X-ray crystallographic studies allowed a profound understanding of the inhibition mechanism^10–13^. As shown in Figure 1, bisulfite binds to Zn(II) in a tetrahedral geometry (Figure 1A), bromide in a distorted tetrahedral geometry (Figure 1B), formate in a trigonal bipyramidal geometry (Figure 1C) and nitrate is one of the few inhibitors non-coordinated to the zinc but binding very nearby to the catalytic metal ion (Figure 1D)^10–14^.

However, up until now, oxygen was never evidenced as a possible ligand of zinc in the CAs, except for one case^15^ which has been poorly understood and less discussed, being obtained from the apo-enzyme which has been reconstituted with diverse metal ions (e.g. Co^2+^). Here we report an interesting finding: when Cu(II) coordinates with His64 (an amino acid residue crucial for the catalytic cycle, as it acts as a proton shuttle between the water coordinated to the zinc and the environment)^16^, oxygen was found bound to Zn(II) within the active site of human (h) CA II, the physiologically dominant mammalian CA isoform^1^.

## 2. Material and methods

### 2.1. Crystallization and X-ray data collection

Crystals of native hCA II were obtained using the hanging drop vapor diffusion method. 2 µl of a solution 10 mg/ml protein solution in water were mixed with 2 µl of a solution containing 2.4 M ammonium sulfate, 50 mM Tris-HC1 pH 8.0, 2 mM HgCl_2_ and were equilibrated against the same solution at 296 K. Crystals grew in two weeks. The metal derivative was prepared by soaking the native crystals in 3 M ammonium sulfate, 50 mM Tris pH 8.0 and 2 mM CuSO_4_ for two days. Crystals were flash-frozen at 100 K using a solution obtained by adding 25% (v/v) glycerol to the mother liquor solution as cryoprotectant. X-ray data were collected at the Centro di Cristallografia Strutturale (CRIST) in Florence using an Oxford Diffraction instrument equipped with a sealed tube Enhance Ultra (Cu) and a Onyx CCD detector. Data were integrated and scaled using the program XDS^16^. Data processing statistics are showed in Table 1.

**Table 1.** Summary of Data Collection and Atomic Model Refinement Statistics.^a^

PDB ID	5EOI
Wavelength (Å)	1.5406
Space Group	P2_1_
Unit cell (a,b,c,β) (Å,°)	42.03, 41.48, 72.07, 104.6
Limiting resolution (Å)	14.8–1.80 (1.91–1.80)
Unique reflections	21538 (2771)
R_sym_ (%)	7.6 (47.8)
R_meas_ (%)	8.4 (61.0)
Redundancy	5.7 (2.4)
Completeness overall (%)	96.1 (78.6)
<I/(I)>	17.12 (1.92)
CC(1/2)	99.8 (69.5)
Refinement statistics	
Resolution range (Å)	14.8–1.8
Unique reflections, working/free	20464/1074
R_factor_ (%)	14.71
R_free_(%)	20.61
No. of protein atoms	
No. of water molecole	344
r.m.s.d. bonds (Å)	0.0184
r.m.s.d. angles (°)	1.884
Ramachandran statistics (%)	
Most favored	95.7
additionally allowed	4.3
outlier regions	0
Average B factor (Å^2^)	
All atoms	16.46
Solvent	30.39

^a^Values in parentheses are for the highest resolution shell.

### 2.2. Structure determination and refinement

The crystal structure of hCA II (PDB ID 4FIK) without solvent molecules and other heteroatoms was used to obtain initial phases using Refmac5^17^. 5% of the unique reflections were selected randomly and excluded from the refinement data set for the purpose of Rfree calculations. Refinements proceeded using normal protocols of positional, isotropic atomic displacement parameters alternating with manual building of the model using COOT^18^. Solvent molecules were introduced automatically using the program ARP^19^. The quality of the final model was assessed with COOT and Rampage^20^. Crystal parameters and model refinement data are summarized in Table 1. Atomic coordinates were deposited in the Protein Data Bank (PDB ID 5EOI). Graphical representations were generated with Chimera^21^.

### 2.3. Computational studies

The structure of carbonic anhydrase in complex with the O_2_ ligand was taken from the present work (PDB ID 5EOI). Standard protonation states were used for all residues. The amber ff99SB-ILDN and GAFF forcefields^22,23^, were employed. The protein was solvated with TIP3P water molecules^24,25^, in a cubic box with smallest solute-edge distance of 12 Å. Cl^−^ counterions were added up to system charge neutrality. The system was locally optimized and equilibrated for 400 ps by classical molecular dynamics (MD) in the NVT ensemble, using the NAMD 2.9 code^26^. Temperature was kept at target value of 300 K by Langevin dynamics with damping parameter set at 5 ps^−1^. The cutoff for the non-bonded interaction was set to 12 Å. Bonds were kept rigid and the integration timestep used was 2 fs. The protein region in the QM box included the whole residues Thr199, Glu106, His96, His94, His119, the Zn^2+^ ion and the ligand O_2_. During classical dynamics equilibration, this region was kept fixed. The system geometry after equilibration was used as the starting conformation for QM/MM (Figure 5a), which was run using the cp2k 4.1 code^27^. All water molecules less than 8 Å away from the Zn^2+^ ion were included in the QM subsystem. The orthorhombic QM box was constructed so that in the initial state the minimum solute-edge distance was larger than 8 Å. Mechanical embedding was used for the QM/MM interface. Goedecker-Teter-Hutter pseudopotentials^28^ and double zeta valence basis sets^29^ with one polarization function were used for all elements. The plane wave cutoff was set to 400 Rydberg. MD simulations were performed in the NVT ensemble, using a timestep of 0.5 fs. The CSVR method^30^ was used to keep the temperature at the target value of 300 K. The system was initially run for 1 ps using a CSVR time constant of 0.01 ps. The time constant was then switched to 0.1 ps for further 1 ps production QM/MM run. Smooth particle mesh Ewald of order 6 and with 1 grid point per Å was used. The BLYP functional^31^ with DFT-D3 corrections^32^ was employed. The carboxylic acid of Glu106 and the catalytic Zn^2+^ were given a formal charge of −1 and +2, respectively. Depending on the formal charge attributed to the O_2_ binder, the total charge of the QM region was −1 (neutral binder) or 0 (charged binder). Average distances and standard deviations were computed using the last 500 fs of the simulations. A smaller full QM model was built by considering only the Zn^2+^, O_2_ and three imidazole binders replacing His96, His94 and His119. Geometry optimization of this model was carried out *in vacuo* using a cubic box with side length of 20 Å, up to a force convergence threshold of 2 · 10^−4^ atomic units. Calculations in the small model were performed at both BLYP and B3LYP^33^ level of theory. The O_2_/Zn^2+^ and O_2_^−^/Zn^2+^ bonding distance differences between the BLYP and B3LYP approach were less than 0.05 Å. Our results for the gas phase model refer to the B3LYP calculations. All QM calculations allowed spin polarization. Orbital decomposition of electronic Kohn-Sham states was obtained by standard projection methods. Quantum theory of atom in molecule (QTAIM) analysis was performed using the Bader^34^^,^^35^ and the Angyan^36^ approach for the charge and bond order estimations, respectively.

## 3. Results and discussion

**Figure 2.**
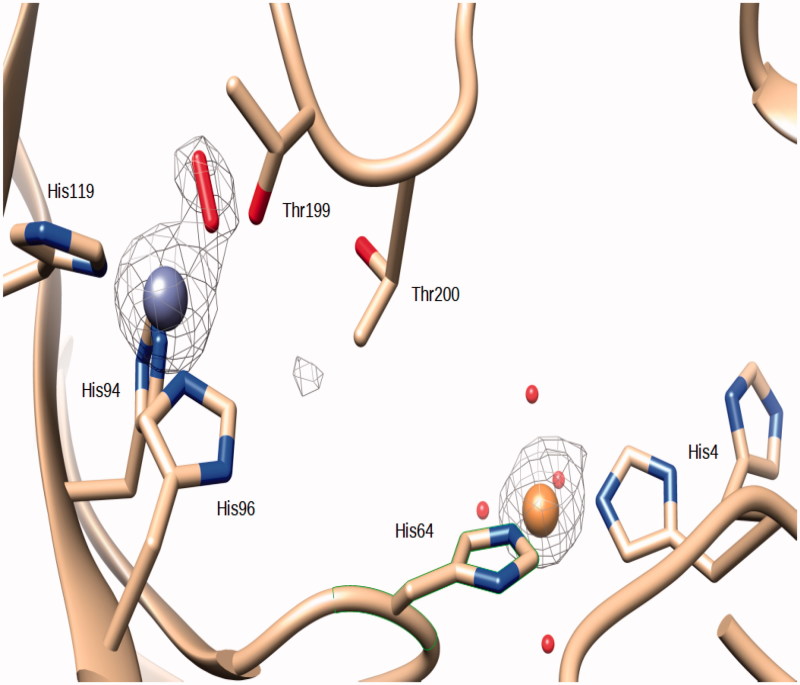
Active site of Zn,Cu-hCA II. The zinc ion (gray sphere) is coordinated by His94, His96 and His119 and a O_2_ molecule. The Cu(II) (orange sphere) bound to His 64 and His4. An omit Fo − Fc electron density map contoured at 3.5 σ level is also shown.

The crystal structure of the Zn,Cu(II)-hCAII complex was obtained from data collected on a crystal of the native enzyme soaked in a solution containing 2 mM CuSO_4_. The initial |Fo - Fc| difference electron density maps showed a spherical density near His64 that was attributed to a copper ion. The Cu(II) ion was introduced at 0.75 occupancy and the B factor refined to a value of 22.3. Two protein residues His64 and His4 coordinate the Cu(II) ion, with two water molecules that complete the coordination sphere around the metal at a distance of 2.2 Å. Two other water molecules are at a distance of 2.5 and 2.8 Å from the Cu(II) ion (Figure 2). Overall, the coordination geometry can be described as distorted octahedral. The distance from the copper ion to the closest (NE2) atom of His64 is 2.1 Å. The corresponding value to His4 is 2.0 Å. The side chain of His4 was modeled in two different conformations. Conversely the side chain of His64, which is actively involved in the proton shuttle and is responsible for converting the Zn-bound water molecule to hydroxide ion^16^, was modeled as a single conformation, whereas it has been often observed to occupy two different conformations as in the first structure of a copper derivative published by Håkansson et al.^15^ In this crystallographic study^15^, the X-ray structures of hCA II metal-substituted derivatives were obtained, in which diverse metal ions replaced the Zn(II) ion in the active site. In the copper derivative, two Cu(II) ions were observed, the second bound at the same N-terminal site reported in the present structure, being coordinated to His64 and His4. No solvent molecules were reported in the second copper coordination sphere^15^. This second copper (II) binding site in hCA II has been recently characterized also thermodynamically and by spectroscopic techniques^37–38^.

In our Zn,Cu-hCAII structure, as in many others, the zinc ion within the catalytic site is coordinate by His94, His96 and His119. An elongated density in the |Fo − Fc| electron density map was present at the position occupied by the zinc coordinated water in the wild-type enzyme (Figure 2). The introduction of a water molecule resulted in residual electron density in the |Fo − Fc| map, but molecular oxygen was successfully modeled into that density (O–O distance refined to 1.2 Å, without applying any restraints, and B-factors to 25.2 and 22.3 for the the two atoms at 1.0 occupancy). O_2_ forms an “end-on” (η^1^) complex with the zinc ion. In “end-on” O2 complexes only one oxygen atom is bound to the metal and they have a bent geometry at the proximal oxygen atom^39^. The coordination of the zinc is tetrahedral, with the oxygen of the O_2_ molecule at a distance of 1.9 Å. The same oxygen is at a distance of 2.6 Å from the OG1 atom of Thr199 and 2.7 Å from a water molecule. The oxygen atom that is not coordinated is at a distance of 2.8 Å from the Zn(II) ion (Figure 2).

**Figure 3.**
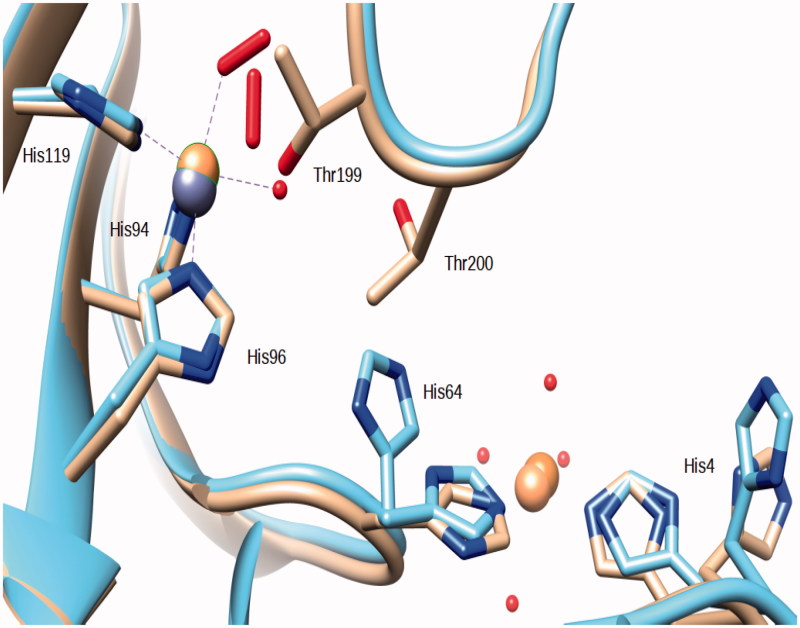
Superposition of the Zn,Cu-hCA II structure (this work) with the copper derivative reported in ref.^15^. Copper ions are represented as orange spheres, zinc as a gray sphere. It should be observed that the O_2_ molecules occupy a different position within the coordination sphere of the two hCA II copper derivatives.

An oxygen molecule bound in the active site of hCA II has already been reported in the structure of the above mentioned copper derivative^15^ and also in the cobalt derivative described in the same article (PDB ID 1RZC and 1RZA). The O_2_ molecule was bound to the Cu(II) and Co(II) ions which replace the zinc in the active site. Contrary to the structure of the Zn,-Cu-hCA II here reported, the Cu(II) and Co(II) ions in the active site maintained also the coordinated water molecule (Figure 3).

**Figure 4.**
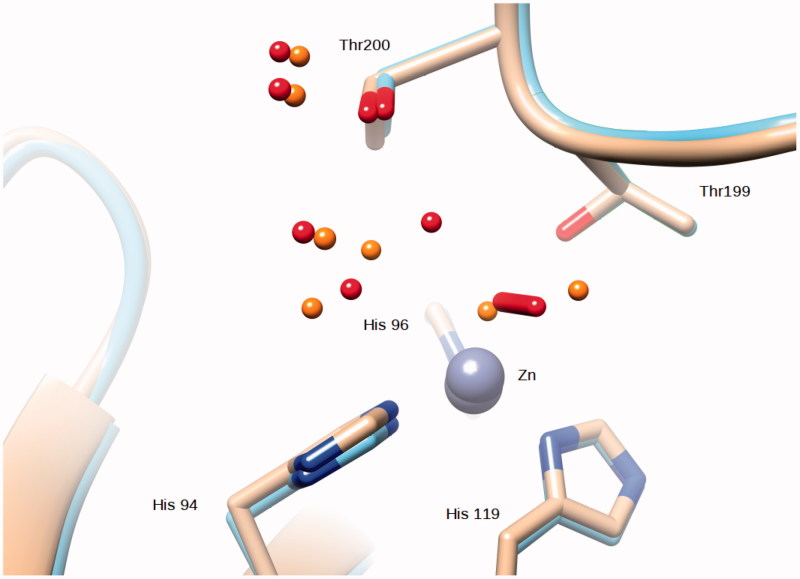
Superposition of the Zn,Cu-hCA II structure (this work) with the native enzyme (PDB ID 2ILI). Zinc ions are represented as gray spheres and water molecues as red spheres (Zn,Cu-hCA II) and orange spheres (native enzyme).

Compared to the native enzyme the O_2_ molecule occupies the position of the zinc-bound water and of the so-called “deep” water (Figure 4).

**Figure 5.**
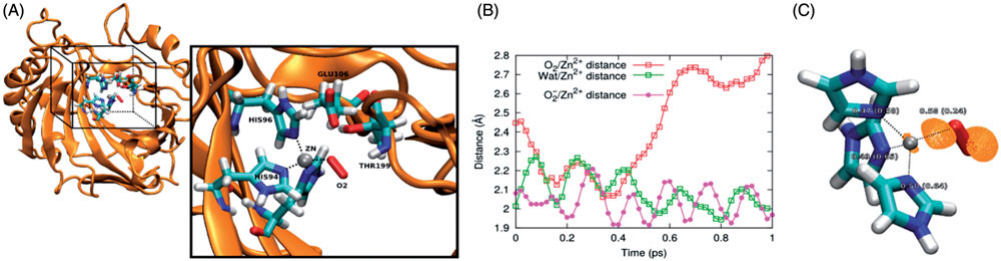
(A) Model used for MD simulations at QM/MM level. Water molecules are included in the calculation but are not shown for clarity. The QM box is reported and QM atoms are explicitly reported in the inset. (B) Time evolution of the O_2_/Zn^2+^, Wat/Zn^2+^ and O_2_^−^/Zn^2+^ distance. Here Wat represents the water molecule displacing O_2_ from the binding site, as described in the main text. (C) Geometry and bonding features of the complex in the small gas phase model. The numbers represent the bond order obtained by the QTAIM approach. Numbers in brackets and without brackets refer to the O_2_/Zn^2+^ and O_2_^−^/Zn^2+^ complex, respectively. The orange surface represents the O_2_^−^/Zn^2+^ complex HOMO density isosurface, computed at a density of 0.05 e^−^/Å^3^.

As oxygen is a rather unexpected CA ligand, and it also does not bind to the enzyme which has not been loaded with copper ions at the N-terminal region, we performed a computational study on this system (Figure 5a). During the molecular dynamics (MD) simulations carried out at the quantum mechanics/molecular mechanics (QM/MM) level, the binding distance between the neutral O_2_ and Zn^2+^ increased from the starting crystallographic value to above 2.80 Å (Figure 5b). Conversely, one water molecule reached a distance of 2.09 ± 0.06 Å from Zn^2+^, displacing the O_2_ molecule from the Zn^2+^ coordination shell. The resulting geometry is reminiscent of the Co^2+^-substituted hCA II^15^ but poorly represents the experimental Zn^2+^/O_2_ coordination. However, MD simulations predict O_2_^-^ to stably bind Zn^2+^ at 2.03 ± 0.04 Å, in good agreement with the crystallographic structure. Similar binding geometries were obtained by structural optimization of the coordination shell in gas phase. In this case, the O_2_^−^/Zn^2+^ and O_2_/Zn^2+^ distances were 1.90 Å and 2.27 Å, respectively. The orbital composition of the HOMO in the O_2_^−^/Zn^2+^ complex (see also Figure 5c) is predominantly O-derived (91%), but also bears a non-negligible contribution from Zn-derived orbitals (7%). This shows that the extra e- added to the system containing neutral O_2_ mainly increases the charge on the O_2_ molecule itself, turning it to a very good extent into O_2_^−^, but also contributes to the covalent character of the resulting O_2_^−^/Zn^2+^ bond. This was also quantified by the quantum theory of atoms in molecule (QTAIM) analysis of the small model, predicting the bond order of the O_2_/Zn^2+^ and O_2_^−^/Zn^2+^ systems to be 0.24 and 0.58, respectively (Figure 5). Overall, computational studies suggest that negatively charged molecular oxygen binding to Zn^2+^ improves both covalent and electrostatic O_2_^−^/Zn^2+^ interactions. Furthermore, from a structural standpoint, the O_2_^−^/Zn^2+^ complex well agrees with the crystallographic outcome, demonstrating that a negative molecular oxygen can better fit into the experimental structure.

## 4. Conclusions

In native hCA II loaded with Cu(II) ions at the N-terminal region, the copper is coordinated by His4 and His64, probably creating a redox center within the active site, which leads to the transfer of one electron to an oxygen molecule which thereafter replaces the water coordinated to the zinc ion deep within the CA active site, becoming a zinc ligand. Although many details of this process are still poorly understood, our data do not preclude the fact that in biological systems copper-loaded CA may have a role in oxygen transport, apart its well-known role in bicarbonate trafficking between the metabolic sites and the excretion organs (lungs and kidneys). In fact, CA II is highly abundant in the blood with almost micromolar concentrations being reached (the hCA I + hCA II concentration in the blood is 0.2 mM^40^ but hCA I is the predominant although catalytically less effective isoform). Future studies are thus warranted to better understand the physiological role of the present finding.

## Acknowldegment

The authors are grateful to Dr. Giuseppina De Simone for Figure 1.
